# Expanding the primary health care workforce through contracting with nongovernmental entities: the cases of Bahia and Rio de Janeiro

**DOI:** 10.1186/s12960-016-0101-3

**Published:** 2016-02-18

**Authors:** Megan Ireland, Luciana Cavalini, Sabado Girardi, Edson C. Araujo, Magnus Lindelow

**Affiliations:** Consultant, Health, Nutrition and Population Global Practice, The World Bank Group, Brasilia, Brazil; Medical Sciences College, Rio de Janeiro State University, Rio de Janeiro, Brazil; Medical School, Federal University of Minas Gerais, Minas Gerais, Brazil; Health, Nutrition and Population Global Practice, The World Bank Group, Washington, DC USA; Health, Education, Social Protection and Poverty in Brazil, World Bank Group, Brasilia, Brazil

**Keywords:** Primary health care, Contracting, Human resources, Health services

## Abstract

**Background:**

Brazil has experienced difficulties in attracting health professionals (especially doctors and nurses) to practice at the primary health care (PHC) level and in rural and remote areas. This study presents two case studies, each a current initiative in contracting for primary health services in Brazil: one for the state of Bahia and the other for the city of Rio de Janeiro. The two models differ considerably in context, needs, modalities, and outcomes. This article does not attempt to evaluate the initiatives but to identify their strengths and weaknesses.

**Methods:**

Analysis was based on indicators produced by the Brazilian health care information systems, a review of literature and other documentation, and key informant interviews.

**Results:**

In the case of Bahia, the state and municipalities decided to create a State Foundation, a new institutional public entity acting under private law that centralizes the hiring of health professionals in order to offer stable positions with career plans and mobility within the state. Results have been mixed as a lower than expected municipal involvement resulted in relatively high administrative costs and consequent default on municipal financial contributions. In the case of Rio de Janeiro, the municipality opted to contract not-for-profit Social Organizations as it made a push to expand access to primary health care in the city. The approach has been successful in expanding coverage, but evidence on cost and performance is weak.

**Conclusions:**

Both cases highlight that improvements in cost and performance data will be critical for meaningful comparative evaluation of delivery arrangements in primary care. Despite the different institutional and implementation arrangements of each model, which make comparison difficult, the analysis provides important lessons for contracting out health professionals for PHC within Brazil and elsewhere.

**Electronic supplementary material:**

The online version of this article (doi:10.1186/s12960-016-0101-3) contains supplementary material, which is available to authorized users.

## Background

Over the last couple of decades, international experience with contracting in the health sector has been growing. Although there are significant differences in context and processes behind these experiences, they have often been motivated by a desire to improve quality and efficiency of service delivery. The “traditional” model of service delivery through public sector providers, with salaried staff on long-term contracts, has been seen as insufficiently responsive or dynamic due to problems of low-powered incentives, lack of management flexibility, and a bureaucratic institutional culture. In contrast, private sector entities are often smaller and unconstrained by the myriad of administrative rules and constraints of the public sector. Hence, at least in principle, contracting can help improve both quality and efficiency by combining management flexibility with more high-powered incentives. Today, both for-profit and not-for-profit providers play an important role in many health systems, in part because of the historic roots of these systems but also as a result of policy decisions to “autonomize” providers or contract with the private sector as a means to improve the performance of service delivery.

As the experience with contracting has grown, so has the associated literature. Part of this literature is concerned with the conditions under which contracting the delivery of public services is likely to be effective, given the uncertainty and information asymmetry that is pervasive in the health sector [3, 4, 6, 14]. There is also a growing body of evidence on specific experiences, including efforts to evaluate to what extent the performance of contracted service providers is superior to other models for delivering services (see, e.g., [10–12, 15, 16]).

The globalization phenomena have changed significantly the organization of health care systems on a global basis. There is a concern about underperformance, and many countries have undertaken reforms as an attempt to remedy those situations. One of the major concerns is related to health care personnel contracting modalities, and experiences are very heterogeneous regarding design, implementation, and outcomes. In summary, the empirical evidence has shown the emergence of three major strategies related to this issue: (1) contractual relations based on delegation of responsibility, including contracts delegating responsibility to private actors, such as contracts for the devolution of a public service; contracts relating to the concession of a geographical area, or public-private partnerships; contracts binding the state and its autonomous institutions, including internal contracting; (2) contractual relations based on an act of purchase, such as the relations between fundholders and health service providers and health service providers’ production processes; and (3) contractual relations based on cooperation, such as weak organizational interpenetration agreements (franchising, collaboration between health care establishments and voluntary associations, or strategic planning at the level of the local health system and health networks) or strong organizational interpenetration agreements (joint management and alliances).

### Brazilian health care system

Brazil formally embarked on its path to universal health care some 25 years ago with the creation of the Unified Health System or SUS (Sistema Único de Saúde). Health care as the right of the individual and duty of the state was written into the 1988 constitution and was the culmination of a broad-based reform process throughout the 1980s that sought democratization and improved social rights. In particular, the “Sanitary Reform Movement” (Movimento da Reforma Sanitária), an informal coalition of health professionals, academics, and other civil society actors, strove for a fundamental break from the prevailing “curative privatizing model” that promoted expanded social security coverage in favor of a “collective public health model” built on the premise of universal access, equity, integrality (comprehensiveness), decentralization, and social participation.

The creation of the SUS unified health financing under one integrated public system that increased and stabilized public financing for health, while at the same time decentralizing the responsibility of service delivery to state and municipal levels. The health system is organized across the three levels of government: federal, state, and municipal. Each level of government has a health fund. At the federal level, the health fund is financed by federal taxes and social security contributions and is a major contributor (45 % in 2009) to the state and municipal funds which are also funded by local revenues. As of 2000, the state and the municipal government are required to allocate a minimum of 12 % and 15 %, respectively, of their overall budgets to health care, although no such provision exists at the federal level [9].

The political decision to reorient and reorganize health care delivery toward a more comprehensive primary health care approach resulted in significant expansion of services, particularly the outpatient network at the municipal level. This was achieved by the creation of the Family Health Strategy (FHS) in 1994, which was designed to expand the coverage of primary health care (PHC) with an emphasis on whole-person care and the social context, and to provide a first point of contact with the broader health system.

In practice, the FHS is based on Family Health Teams (FHT) composed of a doctor, nurse, nurse assistant, and four to six community health workers, organized by geographic regions, with each team providing PHC to around 1000 families (about 3500 people). The teams are either based in Basic Care Units or operate from purpose-built Family Health Clinics that host several FHT. The FHT are expected to provide comprehensive and integrated primary health care to the target population, through services provided at the facility and outreach activities.

Since its launch, coverage of the FHS has expanded rapidly across the country. There are currently over 35 000 FHT, present in 96 % of municipalities, and with an estimated national population coverage of 57 % according to the Department for Basic Care (Ministry of Health, DAB database).^1^ Priority expansion of services into more rural, poorer municipalities, and to poorer communities within them, has enhanced equity of access. Utilization rates have risen across all states and particularly in those with lower levels of income. The FHS has also affected the way Brazilians use public services, reducing the role of hospitals as the “usual source of care” (from 35 % in 1998 to 21 % in 2008) and increasing reliance on PHC facilities (from 42 % to 57 % over the same period) [13]. Several studies have also demonstrated that the FHS has had a significant impact on outcomes, including infant mortality [9].

After a rapid expansion over the first 10 years of implementation, coverage started to stagnate around 2006, in particular in larger municipalities and metropolitan areas. In addition, there has been growing concern with the quality of care. One of the key constraints to expanding coverage and improving quality are the human resource management arrangements in the public sector.

As part of the decentralization of the SUS, municipalities have responsibility for management and delivery of the FHS and are required to adhere to constitutional norms and public law on employment (recruitment, contracting, and payment of professionals) and services. Direct employment options are limited to three types of contracts (as defined by the constitution and administrative law):

- *Public civil servant contracts* are permanent positions that must be contracted through public competitions (merit-based).
- *Temporary contracts* are allowed under circumstances of “exceptional public interest,” the definition of which is contested and malleable.
- *Commissioned positions* are reserved for directorial or high-level managerial functions.

In all cases, levels of remuneration are defined by the municipality. However, salaries are harmonized within municipalities, and there is limited scope for variation across specialization, geographic location, or performance. In addition to the direct constraints on public hiring, other federal laws, such as the Law of Fiscal Responsibility (*Lei de Responsabilidade Fiscal*) which limit municipal spending on personnel to a maximum of 60 % of the municipal budget, also indirectly present barriers to expanding social services. Furthermore, the geographic and sector distribution of medical doctors is marked by inequalities, as many physicians work in urban areas in the private sector or in specialized care. In 2010, 1304 municipalities had a shortage of physicians, especially those in rural, peri-urban, or difficult-to-access areas [5, 8]. There is also an overall shortage of medical doctors with an interest and appropriate qualifications for working in primary care, particularly in underserved parts of the country, due to the relatively lower salaries and the mainly public hiring of primary health care positions, as compared with other specialties. This results in limited attraction for medical graduates to specialize in PHC specialties as there is, for example, a persistent surplus of family health medical residence positions [1].

Given these challenges, many states and municipalities started searching for ways to circumvent public sector rigidities that hampered expansion of primary health care coverage. Contracting out the provision of health services has emerged as one option, and initiatives have been adopted throughout the country.

### New contracting modalities in the health sector

Recognizing the need to increase flexibility in public contracting and service provision, the *Programa Nacional de Publicização* was approved as part of the State Reform process in 1998. The law authorized the transfer of responsibility for running public services and management of public goods and personnel to a specific set of qualified entities, including *Organizações Sociais* or OS, civil society organizations (*Organização da Sociedade Civil de Interesse Público*—*OSCIP*), nongovernmental organizations, philanthropic organizations, cooperatives, and private companies. The objective of the reform was to create a mechanism for facilitating the transfer of certain activities from the state to the private sector. It would be a new form of partnership that called upon the “third sector” (i.e., neither public nor private) to provide services of social interest and public use, but that do not necessarily need to be undertaken by public bodies.

One form of contracting that has emerged as particularly important for the health sector is that of OS. Formally, OS are legal entities under private law, operate on a not-for-profit basis, carry out activities of social value, and operate in partnership with the state. They are primarily financed by public funds and must adopt governance arrangements that allow for state representation. They are subject to public audit (by the *Tribunal de Contas*) and ministerial supervision.

The first experiences with the contracting of OS was in science and technology, with some laboratory services contracted out immediately after the new legislation in 1998. The state of São Paulo was also an early adopter of the OS model as a more flexible alternative for hiring professionals while incorporating private sector management practices, initially focusing on the health sector. The state contracted the management of some hospital services in 1998, and today, OS are involved in most aspects of health service delivery.

Over the last decade, some states in Brazil have also pursued other options for improving the delivery of health services. Specifically, the possibility of using State Foundations started in 2005, when the federal government, through the Ministry of Planning, Budget and Management and the Ministry of Health, and aided by the National School of Public Health and a group of lawyers, began studying broader legal and institutional options for overcoming the rigidities in the health system [7]. State Foundations are decentralized administrative institutions that carry out public activities and provide social services. A key difference between a State Foundation and an OS is that a State Foundation is a public (state-owned) institution, albeit operating under private law (including for contracting and managing staff), whereas an OS is a privately owned institution.

**Fig. 1:**
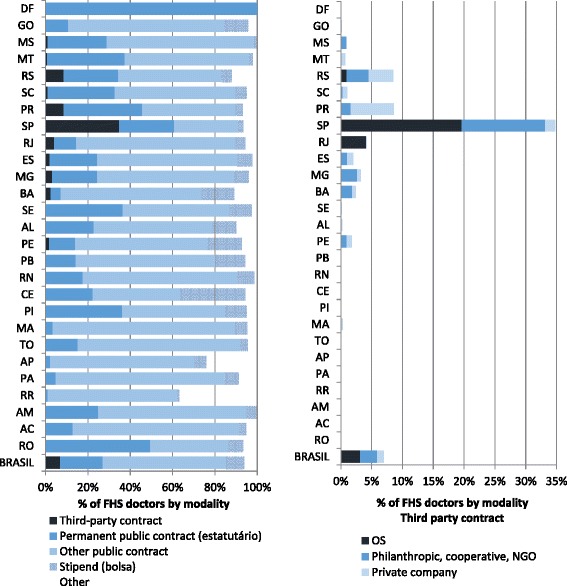
Contracting modalities for doctors in primary care (2013, by state)

Third-party contracting of medical doctors in PHC is still limited, accounting for fewer than 7 % of all contracts, with OS being the most popular alternative (Fig. 1). Permanent public civil servant contracts or other forms of public contract (temporary or commissioned positions) are the predominant form of contracting of medical doctors in PHC (nearly 80 % of all doctors), but irregular forms of contracting through stipends and other means are also significant in many states.

This paper looks at two particularly noteworthy experiences: that of creating a State Foundation (*Fundação Estatal*) in the state of Bahia and contracting with OS (*Organizações Sociais*) in the city of Rio de Janeiro. In both cases, the aim was to expand PHC in underserved areas and improve performance through enhanced accountability and more flexible management, but otherwise, the experiences from the two locations were very different, as were the results (see Table 1 for a comparison of the two models).

**Table 1:** Comparison of contracting models

	Bahia	Rio de Janeiro
When started	2009	2009
Contracting entity	Participating municipalities and the state government of Bahia	Municipality of Rio de Janeiro
Contracted entity/entities	State Foundation	Social Organizations
Stated aims	To formalize and expand employment in primary care and improve quality	Provide primary health services
Main responsibilities of contracted entity	Hiring and training of professional and management support to primary care	Hiring and managing complete Family Health Teams and the services they provide in facilities provided by the municipality
Full-service management	No	Yes
Legal regime	Private law	Private law
Employment regime	Consolidation of Labor Laws (CLT)^a^	CLT
Ownership	Public	Private
Supervision	Office of the Comptroller General^b^	Office of the Comptroller General
Funding	Public	Public

Source: Compiled by the authors

^a^CLT is the main piece of legislation relating to Brazilian labor law and procedural labor law. It was created in 1943, unifying all the existing labor legislation in Brazil. Its main aim is the regulation of individual and collective labor relations. Other labor laws, such as for those working as legal entities (*Pessoa Jurídica*), independent/freelance contract workers, or public civil servants, are covered under a federal statutory legal regime

^b^CGU (Controladoria-Geral da União) or “Comptroller General” is the federal agency responsible for technical supervision, internal control, and public audit

## Methods

The two case studies were chosen for their importance in defining new milestones in human resource contracting for health in Brazil. Although they aim to overcome the same constraints, the two models represent different modalities of contracting and were implemented in different contexts. The selection of case studies was based on the recent trends in the contracting for PHC in Brazil as the OS and the State Foundations are the current dominant modalities of contracting models in the country. The paper does not aim, however, to compare the two experiences given the differences in the contexts, institutional framework, and, particularly, lack of adequate data to control for these differences. The objective is to provide a detailed analysis of each experience to highlight their strengths and weakness that may have influenced the observed outcomes.

This study used a mixed-method study design that included both quantitative and qualitative data collection and analysis. Data collection processes included three stages: firstly, a literature review was undertaken. This review included published literature as well as legislation (laws, decrees, ordinances, resolutions, and operational norms defined by different levels of government). This literature review followed the narrative methodology, searching from scientific publication databases (PubMed, Scopus, and ISI Web of Knowledge) and the gray literature retrieved from searches in generic search engines such as Google. The main search keywords were “primary care” and “contracting.”

Secondly, secondary data on different dimensions of health care provision and outcomes were collected. The secondary data originated from the Brazilian official health care information systems, which are routinely collected, recorded, and stored by the Informatics Department at the Ministry of Health (Departamento de Informática do SUS—DATASUS). Those are the only sources of information that cover the entire period considered in this study, for the indicators chosen. All data were collected from August to September 2013, covering the period 2007–2013.

The third step consisted of in-depth unstructured interviews with key informants from the Secretariat of Health (Rio de Janeiro) and the Secretariat of Health and State Foundation (Bahia). The key informants were recruited based on their participation in the process of defining new contracting rules. As civil servants, with no financial interests in contracting modalities, they were regarded as free from conflict of interest. In the case of the Rio de Janeiro Secretariat, the three informants were part of the team responsible for managing the FHS in the municipality.

Data analysis consisted of (i) qualitative descriptive analysis for the document research and in-depth interviews. Particular attention was paid to management, financing, and service provision issues such as human resources, performance monitoring, and management (For the in-depth interviews, content analysis was carried out by repeated reading of the transcribed interviews in order to codify and analyze the data by theme.) and (ii) quantitative analysis of the secondary data in order to construct a statistical diagnosis of the current state of primary health care in the State of Bahia and the Municipality of Rio de Janeiro.

A complete list of the research themes, indicators, data sources, and type of analysis can be found in Additional file 1.

## Results

### Bahia case study: the State Foundation experience

In January 2007, after an extensive consultative process with stakeholders from the executive and judiciary, as well as civil society, the Bahia State Health Secretariat or SESAB (*Secretaria de Estado da Saúde da Bahia*) proposed the State Foundation as the most appropriate solution for attracting and retaining health professionals that would reduce the proportion of temporary and irregular work contracts and overcome public sector rigidities.

A State Foundation is a state-owned, not-for-profit institution that integrates indirect public administration, but operates within private law, with a mix of private- and public sector governance mechanisms, such as employment contracts. Its legal basis is similar to a state-owned company, except that it functions in the social rather than economic domain, and hence, it may not commercialize its services on the market.

The Family Health State Foundation or FESF (*Fundação Estatal Saúde da Família*) was designed as a strategy for municipalities with the greatest problems in attracting and retaining health professionals and improving the quality of PHC in a coordinated manner. It entailed a tripartite contract between the participating municipalities, the state, and the foundation. Municipal participation was optional, but the benefits of greater stability and quality of services provided were expected to encourage mayors to participate.

The most important function of the FESF was to contract health professionals, in particular doctors, nurses, and dentists, for PHC on behalf of participating municipalities. All recruitment by the FESF was governed by the Plan for Employment, Careers and Salaries (*Plano de Empregos, Carreiras e Salários*), which established public competitive processes and formal labor contracts that allow for mobility across health teams, for career advancement by merit, for continuing education, and for employment stability. This was a pioneering feature of the FESF that was explicitly designed to address the challenges faced by many municipalities in attracting staff by offering both financial and career incentives for professionals to accept positions in underserved areas. The FESF was also expected to provide support to participating municipalities for the management and organization of FHT and the development of primary health care, including training, supervision, and the introduction of management practices supporting quality improvement in primary care.

The FESF was formally created in July 2009. At that time, a total of 256 mayors (61 %) signed Terms of Commitment and Adherence to the FESF and 110 (26 %) proceeded to pass authorizing laws. However, when the contracting process began in September 2009, which entailed signing technical cooperation agreements and management contracts between the municipalities and FESF, only 40 municipalities (10 %) actually contracted the FESF to hire health professionals.

**Fig. 2:**
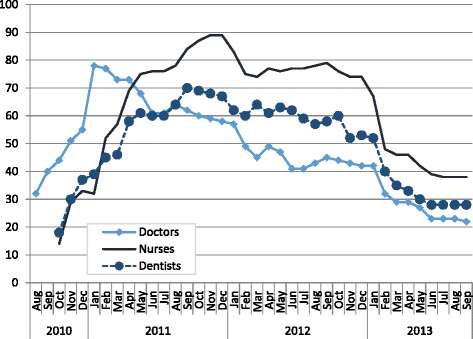
Number of health care workers contracted by the FESF

Of the 40 municipalities contracted in 2009, only 12 (3 %) still had contracts in 2014, accounting for 22 doctors, 38 nurses, and 28 dentists. Due to municipalities’ low participation, the FESF contracted only around 180 FHT, well below the earlier expectation of 1000. The number of staff contracted by the FESF peaked in late 2011 and started declining in mid- to late 2012 (Fig. 2). By late 2012, the default rate on management contracts with municipalities reached 80 % of revenues.

Beyond trying to centralize and coordinate the contracting of health professionals for FHT, the FESF intended to increase the quality of services through various strategies.

First, the professionals hired by FESF were expected to be better qualified for their positions as a result of a more rigorous hiring process. Moreover, the conditions offered by the FESF (stability, social security, career development, etc.) were also expected to help attract qualified professionals.

Second, professionals hired by the FESF underwent 6 months of compulsory (and remunerated) training. All FESF professionals were offered continuing education for specialization or masters programs.

Third, health professionals contracted by the FESF were also offered performance incentives. This was a bonus of 25–50 % of base salary, paid monthly, dependent on meeting the goals set by the FESF. These goals were monitored quantitatively and qualitatively through a dedicated PHC Monitoring System, with payment based on the number of days worked and results.

Fourth, all contracts between municipalities and/or the state with the FESF included targets and goals, to which a 10 % variable portion of payments from municipalities to the FESF was linked. The evaluation of the targets and goals was based on trimestral reports drafted and sent by the FESF to the Monitoring and Evaluation Commission.

### Rio de Janeiro case study: the Social Organization experience

Until recently, health care in the city of Rio de Janeiro relied heavily on an extensive hospital network, including facilities under federal, state, and municipal management, but had very limited primary health care provision. In 2009, the municipality launched the *Saúde Presente* program aimed at expanding FHS coverage. In order to eschew cumbersome public procedures, the municipality constructed purpose-built clinics for several FHT but contracted out the staffing and clinical management to OS. The municipality continued to promote and exercise strategic control over the social actions through entering into management contracts with OS, with which performance targets were agreed to ensure the quality and effectiveness of services.

All contracts between OS and the municipality of Rio de Janeiro were standardized. The contracts contained a defined set of health services that were to be managed, maintained, and equipped with human resources by the OS. The only difference between contracts was the geographic area covered. Contracts were signed for 2 years and were renewable if at least 80 % of the objectives and goals were met. The contracts defined a fixed payment based on number of health teams and an estimate of resources required to cover the Portfolio of Basic Services, as well as a variable, performance-based payment linked to three sets of indicators: incentives paid to the OS based on productivity and quality of service targets, incentives paid to health units based on specific agreed indicators, and incentives paid to health workers. Health units were required to produce trimestral reports relating to agreed indicators.

In May 2009, the law paving the way for the contracting of OS in Rio de Janeiro was enacted and tendering opened immediately. To date, six OS have won tenders to manage FHT in 10 geographical areas. However, only five are still in operation as one of the contracts was canceled due to not meeting minimum standards, and the contract for that territory was passed to another OS. Since contracting with OS started, Family Health Clinics have been implanted all over Rio de Janeiro, primarily by the construction of new clinics (85 new units since 2009) but also by converting Basic Health Units into Family Clinics (49 units that existed before 2009).

The financial reports of the OS were only available as from 2012. They showed a concentration in the hiring of community health agents, doctors, nurses, nurse technicians, and administrators. This was to be expected as the FHS assumes a large number of community health agents working over small territories in direct contact with the families within them. Equally, doctors, nurses, and nurse technicians form the base of FHT, as are administrators that help manage them. The OS did not encounter any problems in attracting professionals. In fact, a critical feature of the OS model was the simplified hiring and the flexibility to pay differentiated (and higher) salaries. The significantly higher salaries for the more qualified positions facilitated hiring and hence created the conditions for the expansion that has been observed.

**Fig. 3:**
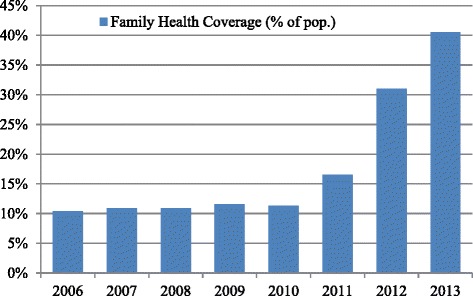
Estimates of FHS coverage, in Rio de Janeiro, 2006–2013

The new Family Health Clinics were successful in rapidly increasing coverage (Fig. 3), in part due to the characteristics of Rio de Janeiro whereby large populations live in favelas. The clinics were implanted in underserved areas with a high population density. They were in most cases large and included multiple FHT and in some cases incorporated laboratories, x-ray facilities, and other services.

The expansion of the FHS in Rio de Janeiro was expected to result in improved coverage of priority interventions (antenatal care, management of patients with chronic diseases, etc.) and, ultimately, improved outcomes. It is still early to assess the extent to which these goals have been achieved. Nonetheless, available data suggest that relative to 2007, the municipality has seen a near doubling of PHC consultations, a large increase in the number of chronic disease patients under active management, and a reduction in avoidable admission from diabetes and associated complications. However, there has only been a modest increase in the share of pregnant women with seven or more prenatal consultations (Table 2).

**Table 2:** Trends in key indicators in Rio de Janeiro municipalities (2007–2013)

	2007	2013	% change
Population	6 178 762	6 429 923	4
Primary health care consultations	3 566 747	6 846 453	92
Pregnant women with 7 or more prenatal consultations	68.5 %	70.8 %	3
Number of diabetics under management	125 317	231 960	85
Number of hypertensive patients under management	544 414	791 951	45
Avoidable admission: diabetes and complications	3.6 %	1.0 %	−71

Source: Official administrative data provided by the Health Secretariat of Rio de Janeiro Municipality

The contracting of OS was also intended to improve performance due to their increased management capacity and flexibility as well as the introduction of performance incentives for both professionals and health facilities. By 2013, however, the performance incentive modality had not operated as planned, since the payment of the variable part of the funding appeared disconnected to achieving the targets: the data available indicate that targets had not been met, while the variable funding had been paid. This was likely to do with problems of information collection rather than poor quality care, and increased reporting of targets in 2013 indicated that reporting was improving. In addition, there also appeared to be distortions with respect to the financing of incentives. The municipality transferred the variable part of the funding to the OS regardless of their performance. The OS, on the other hand, were able to retain these funds if the Family Clinics did not attain their targets and goals. While any retained incentive funds were foreseen to be invested in the health facilities most in need, the fact that the OS stood to capitalize on the poor performance of the Family Clinics they ran represented disincentives for performance and quality.

## Discussion

### Bahia case study

Implementation of the FHS in Bahia started in 1997 and since then has been the principal strategy for strengthening PHC in the state. Since its introduction, FHS coverage has increased continuously, reaching all municipalities and 60 % population coverage by 2011 [2]. Nevertheless, Bahia, like other states in the Northeast, lags behind the rest of Brazil on socioeconomic development, and many municipalities have faced challenges in attracting and retaining health professionals, as well as competition between municipalities for scarce human resources. Moreover, as a result of contractual arrangements, accountability for performance (e.g., complying with the 40 h a week requirement for doctors) has often been weak and there has been little systematic effort to ensure opportunities for continuing education for health professionals in PHC facilities [7]. By centralizing the hiring of professionals for primary health across the state, the FESF intended to create economies of scale and help reduce competition between municipalities and the high rotation of professionals.

There are a number of reasons for the lower than expected participation by municipalities in the FESF. One concerned costs. The fact that FESF offered stable contracts with social security benefits and competitive salaries meant that the cost of hiring professionals through the FESF was comparatively expensive. In addition, the expected economies of scale did not materialize. As a result, administrative costs for participating municipalities were high, and the FESF was not able to achieve bargaining power in the state labor market. Some municipalities were also reluctant to surrender their autonomy to hire and manage their publicly employed health professionals.

One of the main reasons for the decline in participation of the FESF has been the high proportion of municipality-FESF contract defaults. Many municipalities were not transferring funds to the FESF, partly due to a perception that the costs were too high, and yet retained the staff until their contracts were terminated thus defaulting on management contracts. In addition, municipal elections were held at the end of 2012 that changed management in some municipalities, to the detriment of participation with FESF.

Other external factors also impinged upon the appeal of the FESF, such as the simultaneous implementation of other national programs aimed at supplying medical staff to underserved areas. In particular, the Program for the Enhancement of Professionals in PHC or PROVAB (*Programa de Valorização dos Profissionais na Atenção Básica*) that offers a practical 1-year postgraduate course in family health by placing doctors in underserved locations and, more recently, the “More Doctors” program (*Mais Médicos*) that attracts Brazilian and foreign doctors to underserved areas and requires medical students to undertake 2 years in medical residency in PHC facilities as a precondition to graduation, both of which led some municipalities to dismiss FESF-hired professionals in preference for professionals hired under these programs at lower cost.

The impact of the FESF on the quality of primary health care is difficult to assess, in part due to the recent and limited scope of implementation and because contracting with the FESF was voluntary and the participating municipalities are not necessarily comparable to other municipalities. However, the recent performance evaluation of PHC under the National Program for the Improvement in Access and Quality of Primary Health Care or PMAQ (*Programa Nacional de Melhoria do Acesso e da Qualidade*) found that teams with current FESF staff have consistently better ratings. The differences cannot be causally linked to the FESF, but interviewees attributed the better results to the institutional support offered by the FESF to the municipalities [2].

#### The future of the FESF

The FESF model depended on a significant share of municipalities in the state contracting out human resource management in PHC to the foundation, but for a range of reasons, this did not happen, and over time, the number of municipalities doing so declined even further. The leadership of the FESF undertook an administrative and governance reform to reduce administrative costs, seeking to strengthen the focus on quality, productivity, and efficiency by finding a balance between administrative costs for the number of employees and ensuring an appropriate administrative structure for strategic planning and management for results.

In practice, the FESF was obliged to diversify its activities from contracting the workforce to include a contract with the State Health Secretariat-SESAB to hire professionals to develop its home care services linked to the state hospital network, regulatory activities, institutional support to PMAQ, and other services. Hence, although the FESF continues to engage on primary health and work with municipalities, staff for the FHS only accounted for 6 % of employees in 2013, and only 25 % of foundation revenues come from municipalities.

### Rio de Janeiro case study

Rio de Janeiro is the second largest city in Brazil (6 million inhabitants). For many years, the hospital network ensured access to basic care through outpatient departments and emergency rooms, as well as critical inpatient services and specialist care. However, access to the poorer segments of the population has long been problematic and has become increasingly so as the population and health care needs have grown. Moreover, the hospital network does not provide preventive services or health promotion, and integration and coordination of care has been weak.

In light of the remarkable expansion of FHS coverage from around 10 % in 2008 to 41 % in 2013, the contracting of OS can be considered a great success, especially taking into account that many of the Family Health Clinics were constructed from scratch. It can also be said that this reform in the provision of primary health care has succeeded in attracting significant amounts of government funding to the health sector and in particular a huge increase in primary health spending.

Contracting with OS is an interesting model that is based on longer term relationships between the organizations and the municipality. The model requires a high-level of cooperation given that they share responsibilities in primary care, with the municipality responsible for policy and providing the infrastructure, while the OS manage the provision of care and supply of basic drugs and some equipment.

The close relationship has provided conditions for close monitoring and problem-solving and may have contributed to improving performance in ways that are hard to measure. On the other hand, the harder forms of performance accountability envisaged in the contract, in particular performance-based variable payments, still need to be fine-tuned in order to establish a mechanism that delivers on its intended aims and is workable, without inadvertently giving rise to distortions of incentives.

The model has also introduced a potentially problematic dynamic between health workers with regard to compensation. Currently, despite the standardized contracts between the municipality and the OS, there is no apparent link between the resources received by OS per contract and the salaries they pay. In fact, wage inequality exists across the whole primary health network in Rio de Janeiro. Salary disparities exist between public officials assigned to OS and professionals directly hired by them and between professionals hired by different OS but within the same professional category, as well as between professionals working in different geographical locations. OS covering more remote and often poorer areas of the city tend to pay lower salaries. They are therefore more prone to hiring the less qualified, younger professionals for shorter periods of time. Thus, arguably, the introduction of OS has increased the inequality within the primary health system from the traditional management model that had standardized levels of pay. Financial data, however, has only been available since 2012, and therefore, the effects of such wage differentials have yet to be determined but may negatively impact the provision of care.

Although it is probably safe to say that the municipality would not have been able to expand coverage to the extent it has based on the traditional model, some of the gains in terms of quality and effectiveness of PHC may have as much to do with the Family Clinic model (combining multiple teams in purpose-built facilities) as with the OS model. In other words, the ability to hire and manage staff in a flexible way has been an important gain, but the extent to which the contracting out of management of facilities as opposed to simply providing staff is an important aspect of the model is not clear.

There is also insufficient data on costs and efficiency to assess the merit of the OS model relative to alternatives. This is partly an issue of the costs of increased salaries, but also, administrative costs must also be taken into account, both within the OS as well as within the municipality. Assessments of efficiency and performance, however, must take a comprehensive analysis of performance. For instance, higher salary and administrative costs may be justifiable if associated with increased productivity and quality of care.

### Discussion of both studies

In terms of achieving the goals of expanding coverage and improving the quality of PHC services, the picture emerging from the two cases is mixed. The municipality of Rio de Janeiro successfully managed to expand the coverage of primary health care through the FHS. This expansion, in turn, has been associated with a large increase in the utilization of PHC services, which is expected to contribute to improved outcomes over the longer term. Inevitably, a number of implementation issues remain, in particular concerning the collection and compilation of data on costs and performance, which is a critical element of the system of performance-related financial incentives that form part of the contract between the municipality and the OS. At this point, there is limited basis on which to assess the quality of OS performance and to compare services provided by OS with those delivered based on alternative models.

In the case of Bahia, the FESF managed to contract a number of health professionals for participating municipalities, contributing to an expansion of coverage in these locations. However, despite a high level of initial support, the number of municipalities that actually established a contract with the FESF when it was created was very low and has fallen further since. As a result, the intended economies of scale were not achieved, undermining the appeal of the model for municipalities. There are some indications that the FESF has contributed to improved performance where it is operating, but data are limited and by no means conclusive [2].

In assessing contracting experiences and comparing them against other approaches, performance in terms of coverage and quality is important, but the cost implications of the approach also need to be considered. It is often claimed that contracting will improve the technical efficiency of service delivery, although such gains may be partially offset by increases in administrative and transaction costs. However, evidence on these dimensions of contracting experiences if often scant, as is also the case for these two Brazilian cases. There is some limited evidence that the productivity of health providers has increased due to performance incentives and new forms of management. In both models, personnel costs have, however, increased, although this would perhaps have been required in the alternative models as well in order to attract staff. Although there is currently very little information on administrative costs associated with the respective models, it is clear that both the FESF and the OS incur nontrivial administrative costs and there are also transaction costs to be considered in state and municipal administration related to monitoring and oversight of contracts.

Given the differences in the institutional and implementation arrangements, as well as the different socioeconomic context, it is not possible to make a direct comparison between the two models. On the other hand, it is possible to compare with the situation before the contracting arrangements were implemented in Bahia and Rio de Janeiro. In both cases, the experiences managed to achieve expansion of PHC coverage; however, the FESF model did not sustain this expansion given the reduction in the number of municipalities with contracts. The analysis provides some plausible hypothesis to explain this, as, for example, the fact that FESF was more vulnerable to changes in the political scenarios or the loss of autonomy in hiring and managing the workforce by the municipalities contracting with FESF.

## Conclusions

The OS model has a relatively long history in Brazil and is increasingly used for staffing and managing health care provision in different parts of Brazil. Experiences to date have shown that it takes time for the approach to mature, with the need for significant capacity on both the OS and government side (management of services, design of contracts, monitoring of costs and performance, oversight, etc.). Nonetheless, as OS with a focus on health care become more plentiful and experienced, and with continued pressures to contain civil service wage bills at the state and municipal level, the approach will likely continue to grow, in particular in larger municipalities.

The State Foundation model brings some important advantages but has suffered from significant implementation problems. Some of these are related to the complex governance and contracting arrangements that arise between the foundation, the state, and the municipalities. However, the foundation model has also, for better or worse, been undermined by federal initiatives such as *Mais Médicos*, which emerged to address some of the same problems that the FESF was expected to help solve. The future of the foundation model for PHC in Bahia and elsewhere hence depends in large part on how it relates to federal human resource initiatives and on how efforts to diversify into other areas will permit the FESF to maintain a significant focus on primary care.

In both cases, there is a need to carry out rigorous assessments. However, this will require the implementation of reliable and accurate information systems that, as of yet, are not in place. Medium-term indicators are necessary for the measurement of the effectiveness of the system while long-term indicators are needed for the assessment of the impact on the morbidity and mortality of the population covered.

## Additional file

Additional file 1:
**Data analysis of the two case studies.** The file contains a complete list of the research themes, indicators, data sources, and type of analysis

## Footnotes

1 Data from Jan 2014, available at: http://dab.saude.gov.br/portaldab/historico_cobertura_sf.php. Coverage is determined by a person’s residence within the defined catchment area of a Family Health Team.
