# Supplementary material for: Expanding the primary health care workforce through contracting with nongovernmental entities: the cases of Bahia and Rio de Janeiro
Source: Hum Resour Health. 2016 Feb 18;14:6. doi: 10.1186/s12960-016-0101-3 (PMC4758019; doi:10.1186/s12960-016-0101-3)
Supplement: Additional file 1: — Data analysis of the two case studies. The file contains a complete list of the research themes, indicators, data sources, and type of analysis [file 12960_2016_101_MOESM1_ESM.doc]

**Data analysis: indicators, sources and type of analysis**

| **Indicators** | **Data Source** | **Analysis** |
| --- | --- | --- |
| **RIO DE JANEIRO** |  |  |
| **Basic Indicators** |  |  |
| Number of Primary Health Units | Statistical yearbook CEMAPS-RJ, 2013  OSINFO Portal | Time series analysis, 2007-2013 |
| Family Health coverage estimates | Statistical yearbook CEMAPS-RJ, 2013  OSINFO Portal | Time series analysis of proportions, 2007-2013 |
| “Traditional” Primary Health coverage estimates | Statistical yearbook CEMAPS-RJ, 2013  SIA-SUS database | Time series analysis of proportions, 2007-2013 |
| Bolsa Família coverage | *Bolsa Família* database | Time series analysis of proportions, 2007-2013 |
| **Health Expenditure** |  |  |
| Total health expenditure | SIOPS database  Census and estimates  OSINFO Portal | Time series analysis of total expenditures per capita and proportional distribution, 2007-2012 |
| Total primary health expenditure |
| Expenditure on OSs |
| **Process and Results Indicators** | |  |
| Infant mortality rate and its neonatal and post-neonatal components | SIM database  SINASC database | Time series analysis of rates, 2007-2013 |
| Live births, with 7 or more pre-natal visits | SINASC database | Time series analysis of proportions, 2007-2011 |
| Maternal mortality | SIM database  SINASC database | Time series analysis of rates, 2007-2013 |
| Congenital syphilis | SINASC database | Analysis of time series of amounts and incidence, 2007-2010 |
| Smoking among adults | VIGITEL Survey | Time series analysis of prevalence, 2006-2012 |
| Adult obesity | VIGITEL Survey | Time series analysis of prevalence, 2006-2012 |
| Adult sedentariness | VIGITEL Survey | Time series analysis of prevalence, 2006-2012 |
| Adult alcoholism | VIGITEL Survey | Time series analysis of prevalence, 2006-2012 |
| Pap smear coverage in women 25-59 years | VIGITEL Survey | Time series analysis of prevalence, 2006-2012 |
| Mammography coverage in women 50-59 years | VIGITEL Survey | Time series analysis of prevalence, 2006-2012 |
| Avoidable hospital admissions for femoral neck fracture, diabetes and stroke | SIH-SUS database | Time series analysis of rates, 2007-2011 |
| Active follow-up of diabetic patients in primary care | SIAB database | Time series analysis 2007-2013 |
| Active follow-up of hypertensive patients in primary care | SIAB database | Time series analysis 2007-2013 |
| Tuberculosis cure rate | SIAB database | Time series analysis 2007-2013 |
| Dengue deaths | SIAB database | Time series analysis 2007-2013 |
| Percentage of deaths from ill-defined causes | SIAB database | Time series analysis 2006-2010 |
| Access to referral services | SIAB database | Time series analysis 2006-2013 |
| **Social Organization profiles** | |  |
| Number of OSs involved in primary care | OSINFO Portal | Time series analysis 2009-2013 |
| Profile of OSs | Area Management Agreements of SMS-RJ  OSINFO Portal | Qualitative analysis |
| Nature and organization of contracts | Area Management Agreements of SMS-RJ  OSINFO Portal | Qualitative analysis |
| **Financing of Basic Health Units under OS model** | |  |
| Primary care expenditure | SIOPS database  OSINFO Portal | Time series analysis of total expenditures per capita and proportional distribution, 2012-2013 |
| Performance-based financing | OSINFO Portal | Descriptive analysis in three levels: OS units, Basic Health Units and individual averages, January-September 2013 |
| Comparisons between revenue and expenditure as an indicator of autonomy | OSINFO Portal | Time series analysis of income and total expenditure and proportional distribution, 2012-2013 |
| **Primary Health Unit governance** | |  |
| Monitoring of performance target | OTICS website  OSINFO Portal | Qualitative analysis |
| Distribution of health professionals | OSINFO Portal | Time series analysis, 2012-2013 |
| Salary and benefits per position | OSINFO Portal  Municipal legislation | Time series analysis of mean values, 2012-2013 |
| Recruitment and training, duration, and conditions for termination of contracts | Contracts  Interviews | Qualitative analysis |
| **Pharmaceuticals** |  |  |
| Access | Contracts  REMUME list | Qualitative analysis |
| Stock management | OSINFO Portal  Contracts | Qualitative and descriptive analysis |
| **Infrastructure** |  |  |
| Unit characteristics | OTICS website  OSINFO Portal | Qualitative analysis |
| Equipment | OSINFO Portal | Time series analysis, 2012-2013 |
| **BAHIA** |  |  |
| **Basic Indicators** |  |  |
| Primary health coverage | CAMAB – Caderno de Avaliação e Monitoramento da Atenção Básica  (SESAB) | Quantitative Anlysis |
| Number of Health Units | CNES – Cadastro Nacional de Estabelecimentos de Saúde |  |
| **Health Expenditure** |  |  |
| Health expenditure | SIOPS – Sistema de Informaçõs sobre Orçamentos Públicos em Saúde (DATASUS) | Quantitative Analysis |
| **Human Resources** |  |  |
| Human Resources in Heath | CNES - Cadastro Nacional de Estabelecimentos de Saúde do Ministério da Saúde;  RAIS - Relação Anual de Informações Sociais do Ministério do Trabalho e Emprego;  Censo Demográfico do IBGE. | Quantitative Analysis |
| Interviews |  |  |
| Complete range of topics | Current and former State Health Secretaries  Current and former FESF management  FESF-employed doctor  DAB-SESAB  Municipal Health Secretary | Qualitative content analysis |

**Data sources, Rio de Janeiro**

Áreas de Planejamento: http://www.cap10smsdc.com/p/unidades-da-area.html

Bolsa Família: www.mds.gov.br/bolsafamilia

Casa Civil da Prefeitura do Rio de Janeiro: http://www.rio.rj.gov.br/web/cvl/exibeconteudo?id=2806005

CEMAPS-RJ: http://redeoticsrio.org/cemapsrio

Censo Demográfico e estimativas: www.ibge.gov.br

Departamento de Atenção Básica: http://dab.saude.gov.br/portaldab/

Inquérito VIGITEL: http://www2.datasus.gov.br/DATASUS/index.php?area=0207

Lista de Serviços de Saúde: http://200.141.78.79/dlstatic/10112/2624936/DLFE-241101.pdf/Listagemunidadesparaosite.pdf

OTICS: http://redeoticsrio.org

Portal OSINFO: http://www.osinfo.com.br

SIAB: http://www2.datasus.gov.br/DATASUS/index.php?area=0202

SIA-SUS: http://www2.datasus.gov.br/DATASUS/index.php?area=0701&item=1&acao=22&pad=31655

SIH-SUS: http://www2.datasus.gov.br/DATASUS/index.php?area=0701&item=1&acao=25

SIM: http://tabnet.datasus.gov.br/cgi/sim/dados/indice.htm

SINASC: http://tabnet.datasus.gov.br/cgi/sinasc/dados/indice.htm

SIOPS: http://portalsaude.saude.gov.br/portalsaude/texto/7056/908/Dados-Informados.html

SMS-Rio: http://200.141.78.79/web/smsdc/exibeconteudo?article-id=2225015

**Data sources Bahia**

Departamento de Informática do SUS (DATASUS) <http://www2.datasus.gov.br/DATASUS/index.php>

Secretaria de Estado da Saúde da Bahia (SESAB) <http://www.saude.ba.gov.br/>

Microdados fornecidos por meio de cooperação com a Estação de Pesquisa de Sinais de Mercado do Núcleo de Educação em Saúde Coletiva da Faculdade de Medicina da Universidade Federal de Minas Gerais (EPSM/NESCON/FM/UFMG) <http://epsm.nescon.medicina.ufmg.br/epsm/>

Fundação Estatal de Saúde da Família (FESF) [http://www.fesfsus.ba.gov.br](http://www.fesfsus.ba.gov.br/)
